# Size matters: The width and location of a ureteral stone accurately predict the chance of spontaneous passage

**DOI:** 10.1007/s00330-017-4852-6

**Published:** 2017-06-07

**Authors:** Johan Jendeberg, Håkan Geijer, Muhammed Alshamari, Bartosz Cierzniak, Mats Lidén

**Affiliations:** 1 Department of Radiology, Faculty of Medicine and Health, Örebro University Hospital, 70185 Örebro, Sweden; 2 Department of Surgery, Faculty of Medicine and Health, Örebro University, Örebro, Sweden

**Keywords:** Spiral computed tomography, Ureteral calculi, Kidney stone, Ureter, Renal colic

## Abstract

**Objectives:**

To determine how to most accurately predict the chance of spontaneous passage of a ureteral stone using information in the diagnostic non-enhanced computed tomography (NECT) and to create predictive models with smaller stone size intervals than previously possible.

**Methods:**

Retrospectively 392 consecutive patients with ureteric stone on NECT were included. Three radiologists independently measured the stone size. Stone location, side, hydronephrosis, CRP, medical expulsion therapy (MET) and all follow-up radiology until stone expulsion or 26 weeks were recorded. Logistic regressions were performed with spontaneous stone passage in 4 weeks and 20 weeks as the dependent variable.

**Results:**

The spontaneous passage rate in 20 weeks was 312 out of 392 stones, 98% in 0–2 mm, 98% in 3 mm, 81% in 4 mm, 65% in 5 mm, 33% in 6 mm and 9% in ≥6.5 mm wide stones.

The stone size and location predicted spontaneous ureteric stone passage. The side and the grade of hydronephrosis only predicted stone passage in specific subgroups.

**Conclusion:**

Spontaneous passage of a ureteral stone can be predicted with high accuracy with the information available in the NECT. We present a prediction method based on stone size and location.

***Key Points*:**

• *Non*-*enhanced computed tomography can predict the outcome of ureteral stones*.

• *Stone size and location are the most important predictors of spontaneous passage*.

• *Prediction models based on stone width or length and stone location are introduced*.

• *The observed passage rates for stone size in mm*-*intervals are reported*.

• *Clinicians can make better decisions about treatment*.

## Introduction

Urolithiasis is a common cause of acute flank pain with increasing prevalence and increasing costs for health systems [1, 2]. According to earlier studies [3–5] 75–90% of stones in the ureter pass spontaneously. If the stone can be expected to pass spontaneously within a reasonable time and the pain is tolerable, the first approach is watchful waiting, with or without accompanying medical expulsive therapy (MET) [6]. Stones that are not expected to pass are treated with extracorporeal shock wave lithotripsy (ESWL), laser lithotripsy or percutaneous stone extraction via the renal pelvis. There are risks with both the conservative and the invasive approaches, such as sepsis that can occur either because of an obstructing stone or as a post-procedure complication. The main risk of the conservative approach is failure and that the patient has had to endure symptoms to no benefit. The major risk of intervention is that it was unnecessary, exposing the patient to the potential risks of, for example, anaesthesia, upper urinary tract infections and ureteral injury. Thus prediction of the chance for a stone to pass spontaneously is crucial for the appropriate selection of a treatment strategy [7].

It is widely agreed that there is a strong correlation between stone size and location and the likelihood for spontaneous stone passage [3, 4]. More recent studies have suggested other predictive factors, e.g. C-reactive protein (CRP), hydronephrosis [8–11] and side of the stone [12].

There is, however, still no standardized method of stone size measurement, with the most widely used method for diagnosing ureteral stones being non-contrast-enhanced computed tomography (NECT). The uncertainty of stone size measurements includes whether the length or width of the ureteral stone predicts the probability for spontaneous passage. A recent study revealed that 2D measurements underestimate the stone size compared to 3D measurements [13]. Due to the non-standardization of stone size measurement, the meta-analysis published in the 2007 Guidelines from the American Urological Association (AUA) and the European Association of Urology (EAU) have large intervals – 68% of stones <5 mm and 47% of stones >5 mm pass spontaneously [6]. Consequently the guidelines state that watchful waiting is an optional initial approach for ureteral stones <10 mm. These recommendations were not changed in the most recent guidelines from EAU [14].

Thus, the aim of the present study was to determine how to most accurately predict the chance of spontaneous passage of a ureteral stone using the information available in the diagnostic NECT, including reformats in the three most commonly used image planes with clearly defined measurements and to create predictive models for spontaneous stone passage.

## Materials and methods

### Patient population

**Fig. 1:**
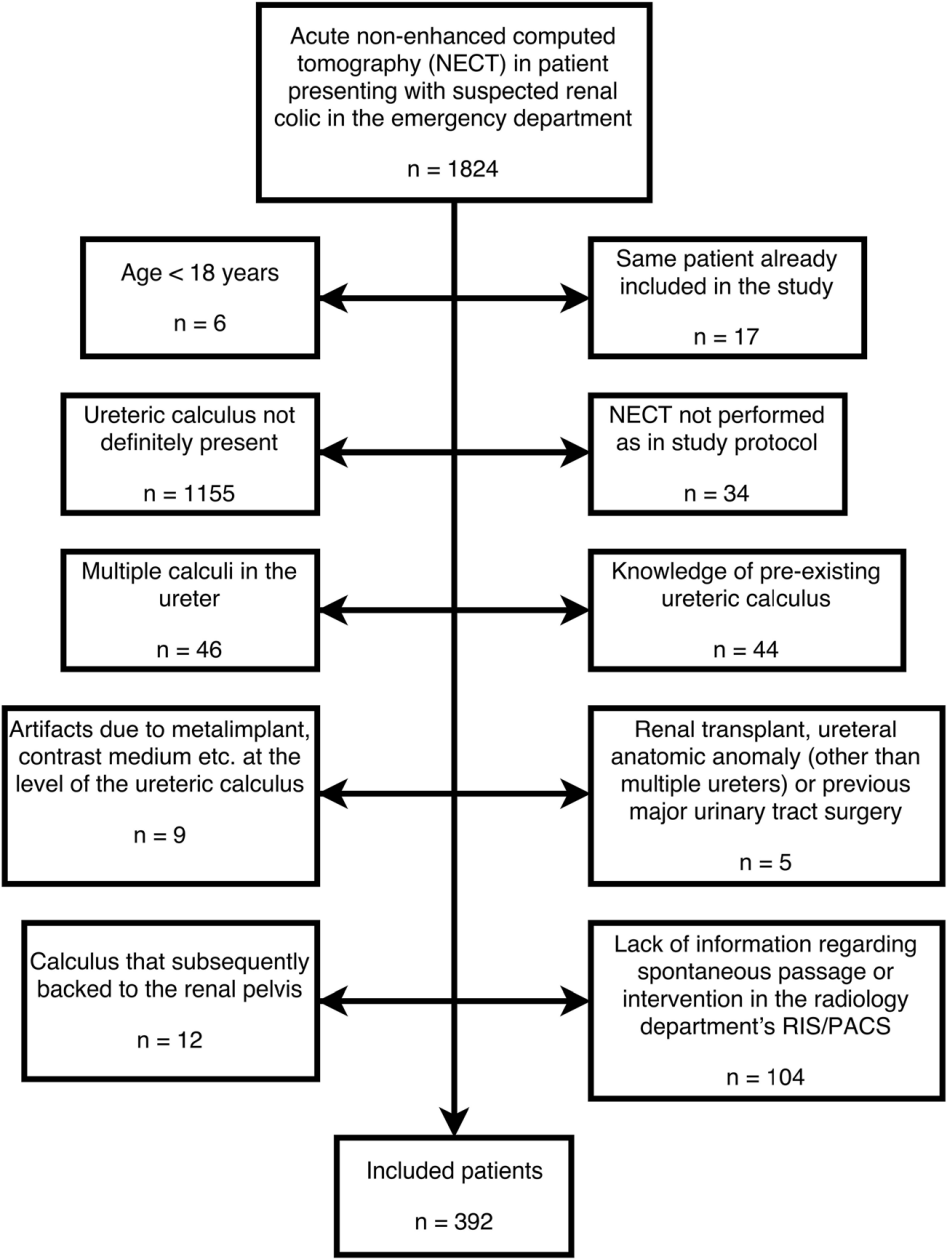
Flowchart showing exclusion criteria and numbers

The Regional Research Ethics Board approved this retrospective study and waived informed consent. We retrospectively reviewed 1,824 consecutive low-dose NECTs performed between April 2012 and September 2014 in patients who presented at our emergency department with suspected renal colic. The sample size of 350–400 patients was estimated with the objective to reach a width of a 95% confidence interval (CI) for the proportion of passed stones of ±10 percentage points. 392 patients were found to have a solitary stone >2 mm (measured in the axial plane) in the ureter and were included in the study. Patient characteristics are shown in Table 1. Exclusion criteria and numbers are shown in Fig. 1.

**Table 1:** Comparison of patients according to spontaneous passage of stone

	All (n = 392)	Short-term outcome		Long-term outcome	
		(n = 220)		(n = 392)	
		(%)		(%)	
		Passage	Non-passage	Passage	Non-passage
		n = 166	n = 54	n = 312	n = 80
Age, y (±SD)	50 (±16)	49 (±15)	52 (±17)	49 (±16)	54 (±16)
Range	18–100	18–84	19–100	18–84	25–100
Sex					
Male	290 (74%)	120 (72%)	43 (80%)	234 (75%)	56 (70%)
Female	102 (26%)	46 (28%)	11 (20%)	78 (25%)	24 (30%)
Side (R to L)					
Right	179 (46%)	73 (44%)	29 (54%)	137 (44%)	42 (52%)
Left	213 (54%)	93 (56%)	25 (46%)	175 (56%)	38 (48%)
Width, mm (±SD)	3.7 (±1.6)	3.2 ± 1. 1	4.9 (±1.5)	3.2 (±1.1)	5.7 (±1.7)
Range	0.5–10.3	0.5–7.7	1.7–10.3	0.5–7.7	1.7–10.3
Location					
Upper ureter	124 (32%)	32 (19%)	29 (54%)	68 (22%)	56 (70%)
Mid ureter	37 (9%)	13 (8%)	8 (15%)	29 (9%)	8 (10%)
Distal ureter	122 (31%)	59 (36%)	11 (20%)	112 (36%)	10 (13%)
UVJ	109 (28%)	62 (37%)	6 (11%)	103 (33%)	6 (7%)
Hydronephrosis					
Grade 3	51 (13%)	11 (7%)	8 (15%)	29 (9%)	22 (27%)
Grade 2	196 (50%)	95 (57%)	21 (39%)	159 (51%)	37 (46%)
Grade 1	107 (27%)	37 (22%)	22 (41%)	88 (28%)	19 (24%)
None	38 (10%)	23 (14%)	3 (6%)	36 (12%)	2 (3%)
CRP mean (±SD)	8.6 (±34)	5.7 (±10.7)	6.0 (±11.1)	7.5 (±34.8)	13.1 (±30)
Range	1–586	1–81	1–65	1–586	1–175
	(11 missing)	(5 missing)	(3 missing)	(7 missing)	
MET	109 (28%)	35 (21%)	16 (30%)	79 (25%)	30 (38%)
	(1 missing)			(1 missing)	
Spontaneous passage	312 (80%)				
Intervention	73 (19%)				
No passage or intervention	7 (2%)				

*Width* stone width (bone window (L300/W1120)), *CRP* C-reactive protein, *MET* medical expulsive therapy

### CT protocol

The CT examinations were performed on two different CT scanners: 166 patients were examined using a 40-detector row CT scanner (Brilliance, Philips Medical Systems, Best, The Netherlands) with a low-dose NECT protocol for the urinary tract (120 kV, 70 mAs/slice, CTDI 4.9 mGy, 40 × 0.625 mm, standard filter [B], supine position). 226 patients were examined with a 2 × 128-channel scanner (Somatom Definition Flash, Siemens, Erlangen, Germany) (120 kVp, 70 mAs/slice CTDI 4.7 mGy128 × 0.6 mm, filter B20f, B25f or I30f, supine position). Three- or 5-mm axial, coronal and sagittal multiplanar reformats (MPR) in the main axes of the patient were generated.

### Image review and patient data

**Fig. 2:**
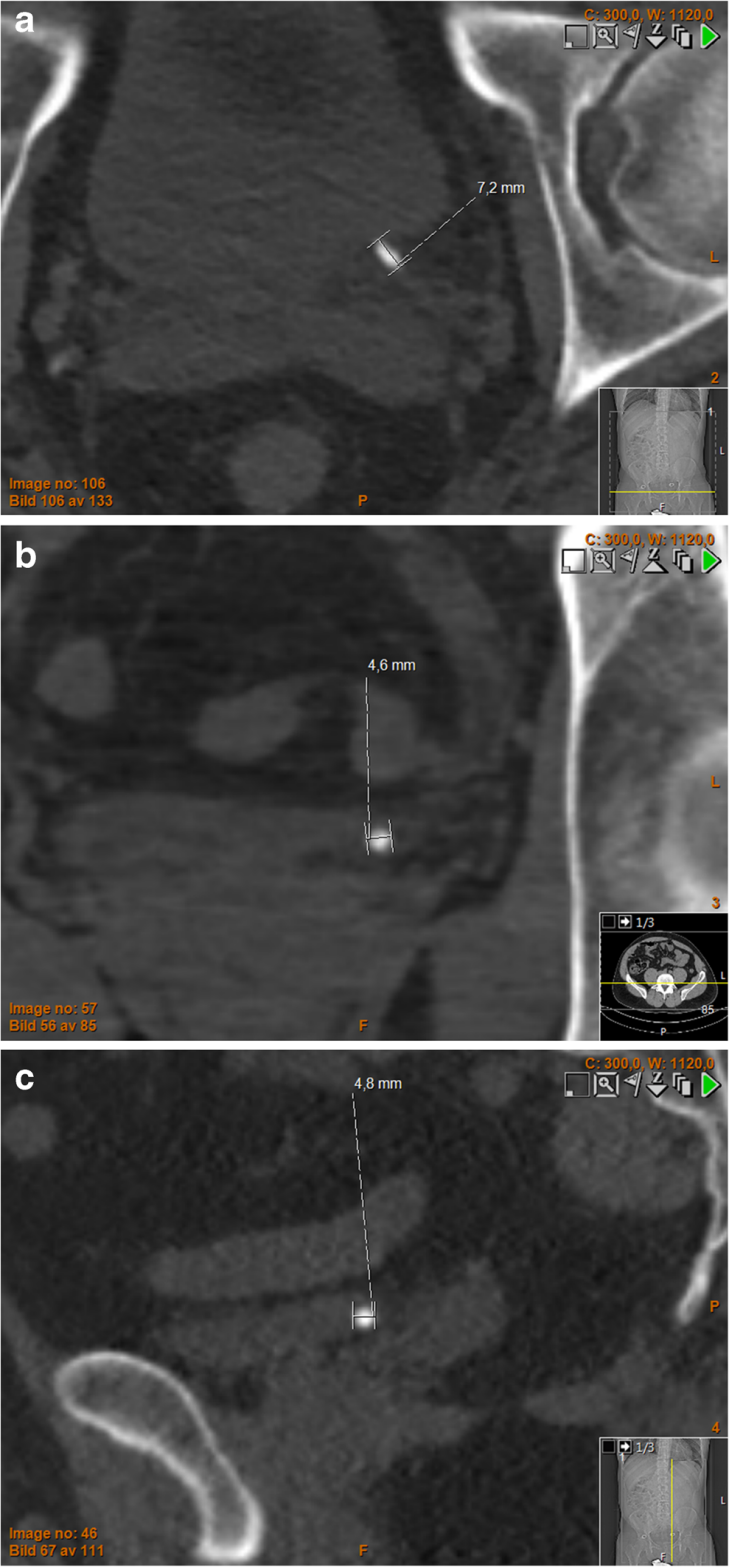
Distal ureteral stone: (**a**) axial, (**b**) coronal, (**c**) sagittal. Non-enhanced computed tomography of the urinary tract, window settings L300/W1120, showing a distal ureteral stone in a 39-year-old male. In each of the three reformats the largest diameter is measured. Stone length = the largest of these measurements (max[ax, cor, sag]). Stone width = the smallest of these measurements (min[ax, cor, sag]). Here the stone length = 7.2 mm and stone width = 4.6 mm

The calculi were independently measured by three radiologists (with 25 (HG), 11 (JJ) and 9 (MA) years of experience each, respectively, in reading abdominal CT) with the integrated PACS measurement tool (Sectra IDS7, Linköping, Sweden). No training for consensus measurement between the readers was performed. The readers were not blinded to the initial report. The largest stone diameter was measured in each of the three reformations (axial, coronal and sagittal) relative to the main axes of the patients’ body, in a standardized bone window setting (L300/W1120) and in a standardized soft tissue window setting (L50/W400) at a fixed zoom level of (pixel to pixel) × 8 [15]. The measurements were reported in mm to 1 decimal point. The length of the calculi was defined as the largest of the three measurements and the width as the smallest (Fig. 2). [13] The mean value of the three readers' estimations was used in the study. If a reader could not see the calculus in one reformation, this was reported as 0 mm.

Stone location was defined as *upper* if the stone was located cranial to the sacroiliac joint, *midureter* (mid) overlying the sacroiliac joint, *distal* to the sacroiliac joint (dist) or at the *ureterovesical junction* (UVJ). Subsequently, the three groups overlying the sacroiliac joint and distal were grouped giving an *upper* and a *lower* (mid, dist and UVJ) position.

The presence of hydronephrosis was graded 0–4 (0 = no, 1 = mild, 2 = moderate, 3 = pronounced, 4 = massive) by reader 1 (JJ) [16].

The side of the stone (right/left), age and sex of the patient were recorded. CRP levels at the time of the primary NECT and whether the patient had been prescribed MET (alfuzosin 10 mg × 1 for 30 days) was recorded from the patient’s medical chart.

### Outcome measure – spontaneous passage of stone

All consecutive radiological examinations in RIS/PACS regarding stone passage and intervention were reviewed up to 26 weeks after the diagnostic NECT. Observed stone passage was defined as presence of follow-up radiological examination (CT or intravenous urography (IVU)) where a ureteral stone was definitely not present anymore. Stone passage was defined as spontaneous if conservative treatment, with or without analgesic or MET, led to stone passage. Any intervention, such as nephropyelostomy, ESWL or ureteroscopy, was recorded as non-spontaneous passage of stone observed at the first day of intervention. The 312 stones with spontaneous passage were verified with IVU (n = 239), NECT (n = 70) or contrast-enhanced CT (CECT) (n = 3).

### Short-term versus long-term outcome

Due to the retrospective nature of the study, the follow-up intervals were non-standardized. Generally the follow-up examination was performed in 4–6 weeks after the first event if watchful waiting was used, irrespective of whether the symptoms resolved or the patient passed and retrieved a stone. In order to achieve an unbiased estimation of the passage rates within approximately 4 weeks, we created a short-term subgroup including patients with the first follow-up examination 28 ± 14 days after the diagnostic NECT. The outcome measure (passed/not passed) in the short-term cohort was obtained from the follow-up examination performed closest to day 28. All patients were included in the long-term follow-up, where the outcome measure was obtained from the follow-up examination closest to 140 days (20 weeks).

### Statistical analyses

The statistical analysis was performed using IBM SPSS for Mac OS v24.0.0.0 (SPSS Inc., Chicago, Il, USA).

Multivariate logistic regression was performed with spontaneous stone passage as the dependent variable and with independent variables as shown in Table 2. Addition of quadratic terms in the multiple regression and visual examination of the predictive curves versus observed passage rates showed no evidence of non-linearity.

**Table 2:** Multivariate logistic regression with all independent variables. Odds ratios (ORs) for stone passage with 95% confidence intervals (CIs)

	Short-term outcome			Long-term outcome		
	OR	95% CI	*p*	OR	95% CI	*p*
Width	0.40	0.17–0.95	0.038	0.31	0.16–0.58	<0.001
Length	0.76	0.40–1.4	0.39	0.86	0.54–1.4	0.54
Position vs. Upper*						
Midureter	2.0	0.49–8.5	0.33	4.3	1.2–15	0.023
Distal	4.5	1.4–14	0.01	7.7	2.7–22	<0.001
UVJ	5.4	1.6–18	0.006	3.3	1.1–10	0.032
Hydronephrosis vs. no hydronephrosis*						
Grade 1	0.21	0.035–1.3	0.087	0.24	0.03–2.2	0.21
Grade 2	1.6	0.27–9.7	0.60	0.60	0.07–5.3	0.65
Grade 3	0.86	0.10–6.9	0.89	0.38	0.04–3.6	0.40
Age	1.0	0.97–1.0	0.92	0.98	0.96–1.0	0.18
Sex (f vs. m)	1.5	0.52–4.6	0.43	0.91	0.39–2.1	0.83
Side (left vs. right)	1.9	0.76–4.7	0.17	2.9	1.3–6.4	0.007
CRP	1.0	0.98–1.1	0.21	0.99	0.99–1.0	0.27
MET	0.94	0.33–2.7	0.91	0.70	0.32–1.5	0.36
Constant	149		0.001	2609		<0.001

*Categorical variable

An OR close to 1 indicates that the variable does not affect the probability of spontaneous stone passage. An OR >1 indicates that this variable is associated with higher probability and an OR <1 that this variable is associated with lower probability of spontaneous stone passage

**Fig. 3:**
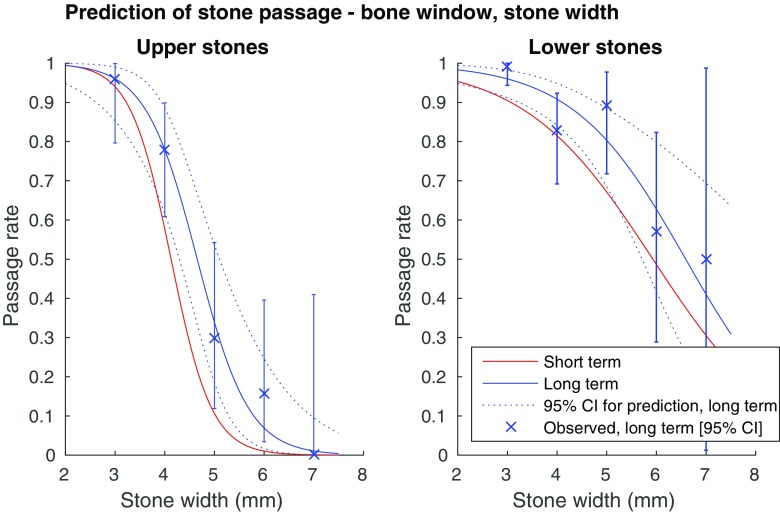
Probability for spontaneous stone passage as a function of stone width, bone window (L300/W1120). (**a**) Upper ureteral stones (univariate logistic regression curves). Error bars showing observed long-term 95% confidence intervals. AUC for the prediction short term: 0.92, long term: 0.93. (**b**) Lower ureteral stones (univariate logistic regression curves). Error bars showing observed long-term 95% confidence intervals. AUC for the short term prediction: 0.80, long term prediction: 0.83

**Fig. 4:**
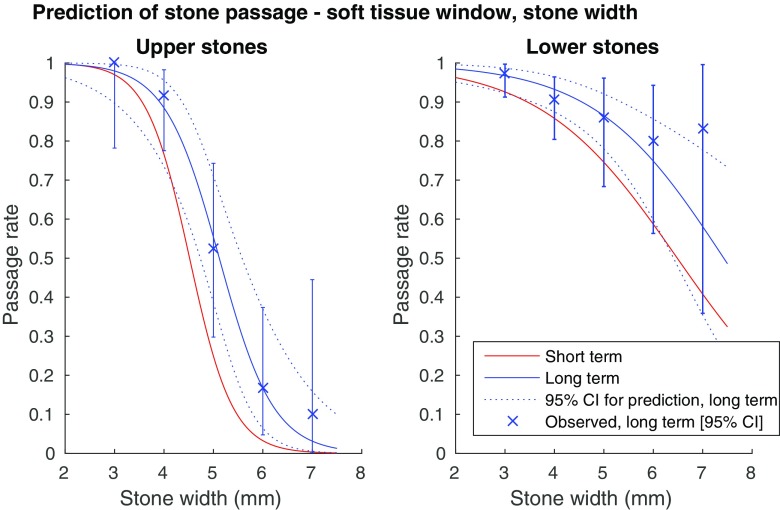
Probability for spontaneous stone passage as a function of stone width, soft tissue window (L50/W400). (**a**) Upper ureteral stones (univariate logistic regression curves). Error bars showing observed long-term 95% confidence intervals. AUC for the short term prediction: 0.92 long term prediction: 0.93. (b) Lower ureteral stones (univariate logistic regression curves). Error bars showing observed long-term 95% confidence intervals. AUC for the short term prediction: 0.81, long term prediction: 0.82

**Fig. 5:**
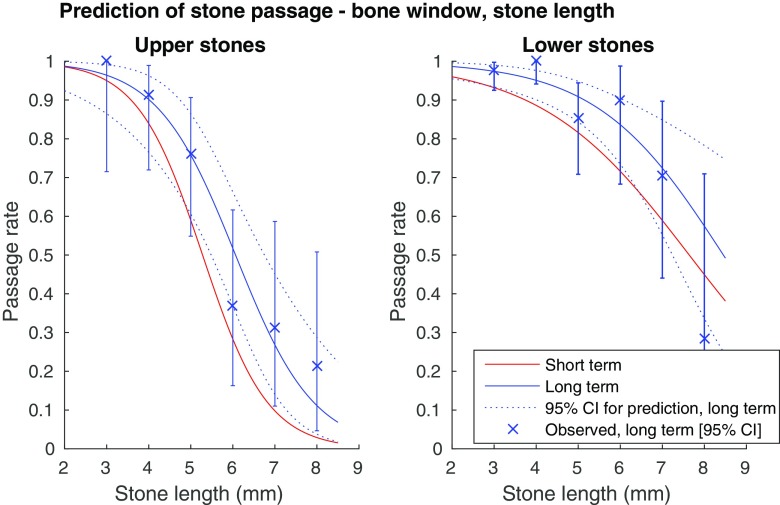
Probability for spontaneous stone passage as a function of stone length, bone window (L300/W1120). (a) Upper ureteral stones (univariate logistic regression curves). Error bars showing observed long-term 95% confidence intervals. AUC for the short term prediction: 0.89, long term prediction: 0.89. (**b**) Lower ureteral stones (univariate logistic regression curves). Error bars showing observed long-term 95% confidence intervals. AUC for the short term prediction: 0.79, long term prediction: 0.83

**Fig. 6:**
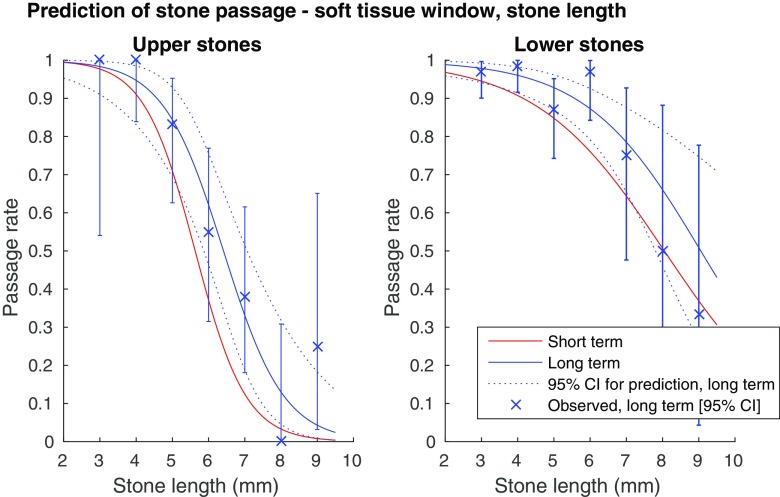
Probability for spontaneous stone passage as a function of stone length, soft tissue window (L50/W400). (**a**) Upper ureteral stones (univariate logistic regression curves). Error bars showing observed long-term 95% confidence intervals. AUC for the short term prediction: 0.90, long term prediction: 0.91. (**b**) Lower ureteral stones (univariate logistic regression curves). Error bars showing observed long-term 95% confidence intervals. AUC for the short term prediction: 0.80, long term prediction: 0.82

Before stepwise regression, collinearity between predictors was assessed with the Spearman correlation coefficient. Because of the high correlation between the stone length and stone width, the length was excluded from the stepwise regression. The other predictors showed a low correlation (|r| < 0.5), and were included in the further analysis. Automated stepwise backward logistic regression was performed in the full cohort and in the subgroups upper and lower stones. Predictive univariate logistic regression curves based on stone width and length were created for the subgroups upper and lower stones in the short and long term (Figs. 3, 4, 5 and 6), for measurements in the bone and soft tissue window separately.

Receiver operating characteristic (ROC) analysis was performed for the predictive regression models.

## Results

Spontaneous stone passage was observed in 312 patients (80%). Intervention was performed in 73 patients (19%) in the 20-week follow-up, mean intervention day 37 (range 0–179). Seven patients without spontaneous passage in 20 weeks did not undergo any intervention during the study period. The mean ±1 SD outcome observation day in the short-term group was 31 ± 7. The observed spontaneous stone passage rate at different stone width, measured in bone window (L300/W1120), and in different locations is listed in Table 3 with 95% CIs for proportions using an exact method. Using the bone window the Bland Altman 95% limits of agreement for the width estimation between reader 1 and 2 was 0.7 ± 1.3 mm, between reader 1 and 3, 0.7 ± 1.3 mm and between reader 2 and 3, 0.1 ± 1.1 mm. Using the soft tissue window the Bland Altman 95% limits of agreement for the width estimation between reader 1 and 2 was 0.8 ± 1.1 mm, between reader 1 and 3, 0.5 ± 1.1 mm and between reader 2 and 3, 0.4 ± 0.9 mm [17].

**Table 3:** Stone passage rate with 95% confidence (CIs) and exact numbers depending on stone width, measured in a standardized bone window (L300/W1120)

	Stone passage rate							
	[95% CI]							
	(passed stones/all stones)							
Stone width (mm)	All stones		Upper stones		Lower stones			
	Both sides		Both sides		Both sides		Left	Right
	Short term	Long term	Short term	Long term	Short term	Long term	Short term	Short term
0-2.4	0.98	0.98	1.00	1.00	0.98	0.97	1.00	0.95
	[0.88–1.00]	[0.92–1.00]	[0,40–1,00]	[0.59–1.00]	[0.87–1.00]	[0.91–1.00]	[0.82–1.00]	[0.76–1.00]
	(43/44)	(82/84)	(4/4)	(7/7)	(39/40)	(75/77)	(19/19)	(20/21)
2.5–3.4	0.92	0.98	0.87	0.96	0.93	0.99	0.89	0.97
	[0.83–0.97]	[0.94–1.00]	[0.60–0.98]	[0.80–1.00]	[0.83–0.98]	[0.94–1.00]	[0.72–0.98]	[0.83–1.00]
	(67/73)	(119/121)	(13/15)	(24/25)	(54/58)	(95/96)	(25/28)	(29/30)
3.5–4.4	0.71	0.81	0.67	0.78	0.74	0.83	0.94	0.50
	[0.57–0.83]	[0.71–0.89]	[0.43–0.85]	[0.61–0.90]	[0.55–0.88]	[0.69–0.92]	[0.71–1.00]	[0.23–0.77]
	(37/52)	(67/83)	(14/21)	(28/36)	(23/31)	(39/47)	(16/17)	(7/14)
4.5–5.4	0.47	0.65	0.09	0.30	0.68	0.89	0.85	0.33
	[0.28–0.66]	[0.49–0.78]	[0.00–0.41]	[0.12–0.54]	[0.43–0.87]	[0.72–0.98]	[0.55–0.98]	[0.04–0.78]
	(14/30)	(31/48)	(1/11)	(6/20)	(13/19)	(25/28)	(11/13)	(2/6)
5.5–6.4	0.21	0.33	0.00	0.16	0.38	0.57	0.40	0.33
	[0.05–0.51]	[0.18–0.52]	[0.00–0.46]	[0.03–0.40]	[0.09–0.76]	[0.29–0.82]	[0.05–0.85]	[0.01–0.91]
	(3/14)	(11/33)	(0/6)	(3/19)	(3/8)	8/14)	(2/5)	(1/3)
≥6.5	0.29	0.09	0.00	0.00	0.67	0.33	1.00	0.00
	[0.04–0.71]	[0.01–0.28]	[0.00–0.60]	[0.00–0.20]	[0.09–0.99]	[0.04–0.78]	[0.16–1.00]	[0.00–0.98]
	(2/7)	(2/23)	(0/4)	(0/17)	(2/3)	(2/6)	(2/2)	(0/1)
All sizes	0.76	0.80	0.52	0.55	0.84	0.91	0.89	0.79
	[0.69–0.81]	[0.75–0.84]	[0.39–0.65]	[0.46–0.64]	[0.78–0.90]	[0.87–0.94]	[0.81–0.95]	[0.68–0.87]
	(166/220)	(312/392)	(32/61)	(68/124)	(134/159)	(244/268)	(75/84)	(59/75)

### Multivariate logistic regression

The multivariate logistic regression analyses were performed using the bone window measurements. The width and length of the ureteral stones were highly correlated (correlation coefficient 0.96). In a univariate logistic regression using width and length as predictor variable the AUCs were similar, 0.90 and 0.89, respectively, for long-term outcome.

In the multivariate logistic regression analysis the stone width took precedence over the stone length, both in short-term (width *p* = 0.038, length *p* = 0.39) and long-term follow up (width *p* < 0.001, length *p* = 0.54) (see Table 2). The problem of collinearity between the predictor variables, which made them unsuitable for simultaneous use in a multivariate model, was solved by removing the stone length in the further analysis.

The stone location was a significant predictor of stone passage. As there were relatively few stones localized in the mid-ureter (37/392, Table 1), and the odds ratio was similar in the mid, dist and UVJ locations (Table 2), the latter three were grouped as lower stones as described earlier. Due to the lack of significant predictive differences between the initially chosen grades of hydronephrosis, this was regrouped into low-grade (grade 0–1) and high-grade (grade 2–4) hydronephrosis in the further analysis.

### Stepwise backwards logistic regression

According to position the stones were divided in the two subgroups, *upper* and *lower stones*.

Multivariate stepwise backwards logistic regression was performed in the full cohort and in these subgroups. In upper stones stone width and side (right vs. left) remained significant predictors of stone passage in the long-term, but in the short-term only stone width was a significant predictor (Table 4).

**Table 4:** Results of stepwise logistic regression with stone width, location (upper vs. lower ureter), side and hydronephrosis (low vs. high grade), age, sex, C-reactive protein (CRP) and medical expulsion therapy (MET) as independent variables. The side remained a significant predictor in the long-term upper ureteral stones and, together with hydronephrosis, in the short-term lower stones. Odds ratio (OR) for stone passage with 95% confidence intervals (CIs)

	Short-term outcome			Long-term outcome		
	OR	95% CI	*p*	OR	95% CI	*p*
*All stones*						
Width	0.28	0.19–0.43	<0.001	0.27	0.20–0.38	<0.001
Location lower vs. upper	4.5	1.9–11	0.001	5.2	2.5–10.7	<0.001
Side left vs. right	2.3	0.99–5.5	0.052	3.2	1.5–6.7	0.003
Hydronephrosis high vs. low grade	4.7	1.9–12	0.001	N/A	N/A	N/A
*Upper stones*						
Width	0.092	0.027–0.32	<0.001	0.13	0.059–0.27	<0.001
Side left vs. right	N/A	N/A	N/A	3.7	1.1–13	0.034
*Lower stones*						
Width	0.33	0.20–0.53	<0.001	0.40	0.28–0.57	<0.001
Side left vs. right	3.6	1.1–11	0.028	2.7	0.92–7.9	0.071
Hydronephrosis high vs. low grade	7.0	2.1–24	0.002	N/A	N/A	N/A
Age	N/A	N/A	N/A	0.97	0.94–1.0	0.052

In lower stones the side and hydronephrosis (low vs. high grade) remained significant predictors of stone passage in the short term in addition to stone width. Left-sided stones had a higher probability of passing spontaneously than right-sided stones. Stones causing moderate to massive hydronephrosis had a higher probability of passing spontaneously than stones causing no or only mild hydronephrosis. In the subgroup long-term follow-up lower stones, only stone width was an unambiguous significant predictor (Table 4). Although not excluded by the stepwise regression, the side and age had *p* > 0.05. Age had an odds ratio of 0.97, indicating minimal influence on the probability of spontaneous passage.

Sex, CRP and MET were not independent predictors of stone passage.

### Predictive logistic regression models

After the stepwise backwards exclusion of the non-significant independent predictor variables predictive univariate logistic regression models were created for the subgroups *upper* and *lower ureteral stones* with the predictor variables stone width and length separately for the bone window and for the soft tissue window. Neither stone side nor hydronephrosis were included in the predictive logistic regression models, due to the low number of stones in each subgroup and consequently broad CIs they would cause and because they were not significant predictors in all parts of the ureter. Furthermore, earlier studies on hydronephrosis and side regarding stone passage have shown divergent results [8, 9, 12, 18].

As seen in Figs. 3, 4, 5 and 6, there is a steep middle part of the logistic regression model curves, especially for the upper stones, making the estimated probability for passage range from about 80% in a 4-mm wide stone, to about 10% in a 6-mm wide stone in the long-term follow-up bone window.

## Discussion

Ever since NECT replaced KUB in the 1990s as the primary tool for diagnosing ureteral stones there have been controversies concerning how to measure the ureteral stones for prediction of stone passage rate. A pivotal aspect has been the diversity and sometimes lack of definitions of stone width and length [5, 10–12, 15, 19–26].

We created logistic regression models for prediction of spontaneous passage using a clear definition of the stone width and length. The strong correlation between the width and the length of a ureteral stone suggests that either measurement can be used in a predictive model with a similar AUC. Thus the choice between the width and the length as a predictor variable is less important, while it is of utmost importance that the selected measure is used consistently. Although the stone width took precedence over the length in the multivariate regression and measuring the width conforms to the intention in earlier studies, we provide predictive models for both stone width and length (Figs. 3, 4, 5, and 6) [3, 5, 18]. We also recommend a high level of magnification to be used, together with a predefined window setting.

To the best of our knowledge the present study with spontaneous passage as the outcome has included the largest number of ureteral stones since the start of the CT era, which made it possible to create logistic univariate regression models for prediction of passage probabilities given the stone size for different stone locations and with two different window settings of L300/W1120 and L50/W400.

It is widely agreed that the stone location is an important predictor of stone passage [3–5, 12]. Our results suggest a classification of stone location into *upper* and *lower* ureteral stones when predicting stone passage.

We demonstrated that left-sided ureteral stones seem to pass significantly more often than right-sided in some analyses (see Table 4). Sfoungaristos et al. suggested that the reason might be that the right ureter is typically adherent to the peritoneum, in contrast to the left ureter, providing a better peristalsis in the left ureter [12].

In the present study lower stones causing moderate to massive hydronephrosis passed significantly more often within 4 weeks than stones causing no or only mild hydronephrosis. In the long term there was no significant difference between the grades of hydronephrosis.

The results of earlier studies on hydronephrosis and side regarding stone passage are divergent [8, 9, 12, 18]. Furthermore the CIs became very broad when hydronephrosis and side were added to the predictive regression models, and we chose not to include either of these variables in the models.

In contrast to the results of previous studies [8, 10, 11], CRP was not an independent predictor of spontaneous ureteral stone passage in our study. We did not find MET to be a significant predictor of stone passage, but our study was not designed to assess the impact of MET on stone passage. Only 29% of the patients were prescribed MET and the study should primarily be considered as conducted without MET. Until most recently, there seemed to be convincing evidence [27, 28] that MET would facilitate stone passage, but this was contradicted by a large recently published randomized controlled trial [25] that did not find any difference between MET and placebo.

Our study has limitations. As the study was retrospective the clinical management of the patients affected the observations of spontaneous passage. This effect was reduced in the short-term analysis by selecting a subgroup where the first observation of stone status was within approximately 4 weeks. Furthermore, the bone window setting is sensitive to reader variations due to a large part of stone with indeterminate grey-scale intensities in the periphery, and the reader variations were large even in the soft tissue window. The variability in the study was reduced by using the mean value taken from three readers. However, the reader variations of any radiologist applying the results will affect the estimated prognosis for the individual patient.

As the study was made retrospectively, the follow-up examinations were not standardized. Conforming to the clinical routines of our urology department, most of the follow-ups were IVUs. Very small or low-density stones that caused no obstruction could possibly be missed using IVU. However, all subsequent radiological examinations were checked for possible missed stones with a follow-up time of each patient of at least 6 months and the possibility for misclassification of stone passage based on IVU was therefore considered to be low.

The steep middle part of the predictive regression curve simplifies the decision of treatment strategy based on stone size, but is also sensitive for inter- and intra-observer measuring differences and variances related to scan parameters. Patel et al. showed that interobserver variability could be substantially reduced with an automated volume measurement. Several different promising automated reader-independent measurement methods [15, 29–31] have been proposed and further studies with these methods would be of interest.

In conclusion, our results show that spontaneous passage of a ureteral stone can be predicted with high accuracy with the knowledge of stone width or length and whether the stone is localized cranial to the sacroiliac joint or not, if standardized window settings and magnifications are used. The present study demonstrates a method for predicting the probability for stone passage in the short- and long-term, based on stone size and location.

## Abbreviations

AUA: American Urological Association
AUC: Area under the curve
CRP: C-reactive protein
EAU: European Association of Urology
ESWL: Extracorporeal shock-wave lithotripsy
IVU: Intravenous urography
KUB: Kidney-Ureter-Bladder abdominal radiography
MET: Medical expulsion therapy
MPR: Multiplanar reformats
NECT: Non-enhanced computed tomography
PACS: Picture archiving and communication system
RIS: Radiology information system
UVJ: Ureterovesical junction
